# Case report: Diagnostic challenges in a patient with alcohol use disorder that developed following a stroke

**DOI:** 10.3389/fpsyt.2023.1116922

**Published:** 2023-04-12

**Authors:** Jacob Zeitlin, Nabil Kotbi

**Affiliations:** ^1^Weill Cornell Medical College, New York, NY, United States; ^2^Addiction & Substance Use Rehabilitation, Westchester Behavioral Health Center, White Plains, NY, United States

**Keywords:** case report, substance use disorder, delirium, coping strategies, polypharmacy

## Abstract

Patients with comorbid neurological and psychiatric diseases often face considerable impairment, causing challenges that pervade many aspects of their lives. Symptoms can be especially taxing when one or more of these conditions is severely disabling, as the resulting disability can make it more challenging to address comorbidities. For clinicians, such patients can be quite difficult to both diagnose and treat given the immense potential for overlap between the underlying psychiatric and neurologic causes of their symptoms—as well as the degree to which they might exacerbate or, conversely, mask one another. These intricate relationships can also obscure the workup of more acute pathologies, such as alcohol withdrawal and delirium. This report details the complex history and clinical challenges in a 54-year-old man who was no longer able to work after developing multiple neurologic deficits from a left MCA stroke a decade earlier. The intellectual and motor disabilities he faced in the aftermath of his stroke were subsequently compounded by a steady increase in alcohol consumption, with his behavior ultimately progressing to severe alcohol use disorder. The coinciding neurologic and psychiatric manifestations obfuscate the workup—and therefore the management—of his major depressive disorder. In pursuit of the optimal approach to address these comorbid conditions and promote recovery, an investigation into possible mechanisms by which they are interconnected revealed several potential neuropsychiatric explanations that suggest targets for future therapeutic strategies.

## 1. Introduction

Following a stroke, patients can develop behavioral changes due to a variety of underlying mechanisms. Lesions in specific anatomic regions of the brain have well-documented stereotypical manifestations that might explain some of these behaviors (1). Behavioral changes can also develop as coping mechanisms to deal with the wide array of psychosocial stressors that arise after a large stroke (2, 3). While these disparate pathophysiological processes may result in the same ultimate behavioral consequences for patients, they warrant scrutiny from a clinical perspective. It is crucial to determine if one mechanism or another is predominantly contributing to their symptoms, if the changes can be altogether better explained by an alternative unrecognized mechanism, and how these processes might interact with one another. Not only does the investigation of these questions allow clinicians to gain a more comprehensive understanding of their patients, but it can also potentially improve outcomes, as the optimal therapeutic approach is often influenced by the predominant underlying pathophysiology (4). To complicate this further, patients with large strokes often present with additional neurologic deficits (e.g., aphasia) that pose nuanced diagnostic challenges. This report describes the case of a 54-year-old man who developed an alcohol use disorder—on top of other complications—over the decade following a left MCA stroke. The purpose of this case report is to illustrate the nature of these diagnostic challenges and to suggest a strategic framework for tackling similarly complex cases.

## 2. Case description

The patient of interest is a 54-year-old man with a history of left MCA stroke, type 2 diabetes, alcohol use disorder, and major depressive disorder. He has been hospitalized multiple times for clinical monitoring of alcohol withdrawal. Most recently, this patient presented to the emergency department at an outside hospital with symptoms of alcohol withdrawal including tremors, diaphoresis, and anxiety. After completing alcohol detoxification, he began inpatient rehabilitation for alcohol use disorder.

Prior to his stroke, this patient had been a “social drinker,” consuming about 2 drinks per week. He relates that alcohol was not interfering with his life. That year, however, he suffered a left MCA stroke, resulting in persistent residual deficits that include right-sided weakness, expressive aphasia, and mild cognitive impairment. Over the course of the next several years, he gradually began drinking alcohol more heavily. He reports the problematic drinking started after he left his intellectually demanding occupation and began collecting disability benefits due to the cognitive impairment. Furthermore, he was unable to engage in other activities he had previously enjoyed, such as running, due to residual right-sided weakness throughout his body. Then, a close family member of his died of a myocardial infarction. In the years since the stroke, he endorses becoming “socially withdrawn” and feeling “useless,” which have contributed to a chronically depressed mood and an inability to “enjoy life as [he] used to.” Also during this timeframe, his diabetes progressed, and the patient began treatment with insulin. This progression was associated with new-onset peripheral neuropathy, which required daily gabapentin. Eventually, his drinking increased to a pint of vodka daily, leading numerous consequences in his life including medical and relationship difficulties.

**Figure 1:**
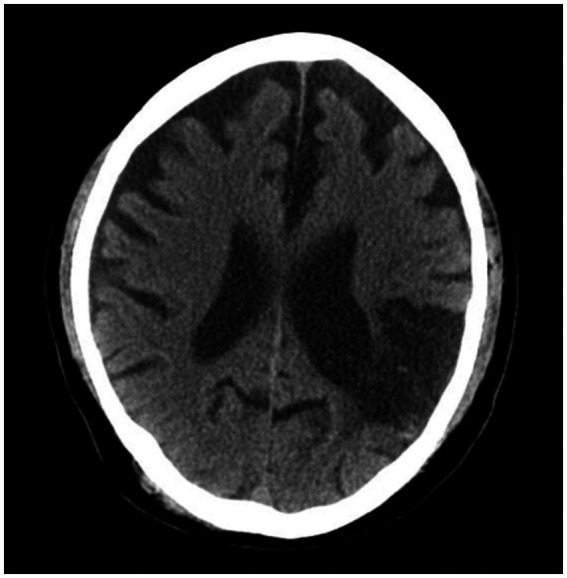
Non-contrast CT scan obtained from outside hospital.

With treatment, he was able to maintain sobriety for a year before a fulminant relapse, again drinking a pint of vodka daily, which led to an inpatient rehabilitation. At that time, he was discharged on oral acamprosate and long-acting injectable naltrexone. However, he began drinking again, ultimately presenting with acute withdrawal to an outside hospital to make another attempt to address his alcohol use disorder. While in the hospital, medical workup was unremarkable. A non-contrast CT scan of the head (Figure 1) was ordered and showed no acute changes. Chronic changes, which had previously been observed on CT, included the following:

1. There is moderate, diffuse ventricular and sulcal prominence consistent with volume loss, greater than expected for age.
2. There is a large region of encephalomalacia in the left posterior frontal and parietal lobes in a left MCA distribution. There is indistinctness of the gray-white interface in the affected region.

Alcohol withdrawal was managed with a symptom-triggered regimen of IV lorazepam and oral chlordiazepoxide according to the Clinical Institute Withdrawal Assessment for Alcohol (5). After 24 h without requiring benzodiazepines, he was admitted for the current short-term rehabilitation.

Upon admission to the inpatient rehabilitation facility, the patient exhibited intermittent states of confusion without agitation or aggressive behavior. In addition to his baseline expressive aphasia, mental status examination was notable for not knowing where he was, increased distractibility, and disorganized speech. He also became visibly frustrated when asked to name the current president and the date and said he was “confused because everyone keeps asking that question.” Staff members were in agreement that this mental status was an obvious and abrupt deterioration from his baseline during the recent admission over the summer. Therefore, the clinical team obtained collateral history from his wife to confirm chart review of his medical and psychiatric history, including alcohol use and premorbid functioning. On physical exam, he also had a gaze-evoked left-beating nystagmus. Given concern for possible Wernicke encephalopathy in the setting of chronic alcohol use, IM thiamine was administered (to augment the standing oral thiamine started during his detoxification). Several days later, his symptoms remain unchanged.

## 3. Discussion

This case highlights multiple meaningful, thought-provoking diagnostic challenges. One such challenge is establishing the most plausible explanations for altered mental status in this patient. Acutely altered mental status has numerous possible etiologies, some of which can be especially difficult to rule out given the challenges associated with taking the history of an altered patient. With the acute onset of disorientation and inattention, the patient likely experienced hypoactive delirium, a condition that is typically secondary to medical complications and/or medications.

Upon further inspection of his outside hospital course immediately prior to admission, it was discovered that while no benzodiazepines had been administered in the final 24 h of his hospitalization, he had been given a total of 800 mg of chlordiazepoxide over the 24 h leading up to his final dose. While this might have been sufficient to induce delirium even in a younger and healthier patient, the patient in this case has a multitude of risk factors that make him susceptible. Some of these factors include hospitalization, history of stroke, alcohol use disorder, alcohol withdrawal, depression, diabetes, and polypharmacy, among others.

While the benzodiazepines are likely a contributing factor, it is necessary to consider additional etiologies and exacerbating circumstances that are possibly complicating his delirium, and life-threatening metabolic disturbances must be ruled out. Wernicke encephalopathy, for which chronic alcohol use is a major risk factor, presents with confusion, oculomotor dysfunction, and gait ataxia in the setting of thiamine deficiency. This condition, however, is less convincing of an etiology in the setting of daily thiamine supplementation during his prior hospitalization. Rather, it is more likely that the nystagmus is an incidental exam finding caused by a physiologic end-point nystagmus.

Another cause of delirium is polypharmacy. In addition to benzodiazepines, he is on a medication regimen that consists of metoprolol, aspirin, gabapentin, duloxetine, atorvastatin, acetaminophen, naltrexone, acamprosate, and several others. Gabapentin, for example, may have increased the risk of withdrawal delirium. Furthermore, there is a lack of strong evidence to support the use of antidepressants, such as duloxetine, to treat depression in patients with alcohol use disorder. Regardless of whether polypharmacy was an underlying etiology of the delirium, his extensive medication list warranted review to assess for drug–drug interactions and to determine if any medications could be eliminated or reduced. When managing patient cases with similarities to this one, close review and verification of medication lists is imperative.

In addition to analyzing the complex nature of his delirium, a second fascinating issue in this patient is the conceivable connection between his stroke and the ensuing onset of his alcohol use disorder. In the aftermath of a severe stroke such as the one this patient experienced, there are several possible explanations for the development of a new-onset alcohol use disorder. An investigation into the predominant neuropsychiatric mechanism is crucial to guide the overarching therapeutic approach. Plausible mechanisms to explain the change in behavior after his stroke include maladaptive coping mechanisms in the setting of new psychosocial stressors (2, 3), cerebrovascular tissue injury leading to impaired neurological functioning (1, 6–8), and decompensation of a previously well-masked psychiatric illness in the setting of his stroke (9).

The chronic depression that the patient endorses is a common symptom in patients who have survived a stroke: the prevalence of depression at any time after experiencing a stroke is estimated to be 29 percent (2). This rate was shown to be significantly elevated in patients with some of the risk factors seen in Mr. A, including greater degrees of disability, cognitive impairment, and stroke severity. Mr. A suffered a significant decrease in functional status following his stroke, never returning to work afterwards. On an individual level, these circumstances were compounded by the fact that 2 years after the stroke, a close family member died. This highlights the importance of obtaining detailed histories in patients with presentations similar to that of Mr. A, as this might help to capture psychosocial stressors and other complicating patients factors that can be addressed by the care team.

Previous research has also suggested that patients with depression after a stroke have different attitudes on coping strategies than do patients without depression: in their responses on a 20-item checklist, patients with depression expressed decreased levels of both rational cognitive appraisal (i.e., taking things “one step at a time”) and behavioral action (i.e., taking “positive action to regain strength”) (3). This finding, which might be explained by underlying patient attributes, is insufficient to establish causation. It does, however, convey prognostic utility, establishing that this patient is at higher risk of displaying an attitude that promotes maladaptive coping strategies in response to psychosocial stressors. This should be taken into consideration for patients like Mr. A, in whom appropriate education regarding coping mechanisms is crucial.

In contrast to these psychological explanations, an alternative pathophysiology underlying alcohol use disorder in this patient might be that it developed as a direct sequela of his initial ischemic insult. Cognitive deficits of varying severities are seen in about 46 percent of patients 6 months following ischemic stroke (6). Consequently, male stroke patients between the ages of 18 and 50 are 3.2 times more likely to become unemployed within the first 8 years after a stroke compared to healthy peers (7). In the general population, cognitive impairment and unemployment are both independent risk factors for alcohol use disorder (8).

Other recent neuropsychiatric research has demonstrated that global cortical thinning, a common finding in patients who have had strokes, is associated with an increased risk for alcohol use disorder (1). As was noted on his most recent CT scan (Figure 1), his brain demonstrates diffuse volume loss as well as a large region of chronic encephalomalacia. While this isolated finding does not provide a complete picture of how (if at all) parenchymal damage contributed to the alcohol use in this specific individual, this literature does highlight the concept that structural consequences following a stroke likely play a role in modulating behavioral changes down the line.

The association of alcohol use disorder with comorbid mood disorders has been described in multiple large cohort studies. Using data from the National Epidemiologic Survey on Alcohol and Related Conditions, one study quantified the degree to which mood disorders correlated with lifetime risk of alcohol use disorder (9). The study elucidated that the odds ratio of having a history of any mood disorder was 2.4. While bipolar I disorder was the largest risk factor among the individual mood disorders, major depressive disorder was another independent risk factor for alcohol use disorder, with an odds ratio of 1.9.

Extrapolating to this patient, it is possible that he suffered from an undiagnosed mood disorder, such as major depressive disorder, before the stroke. If that were the case, the stroke and its associated stressors would likely have exacerbated his symptoms, ultimately contributing to his alcohol use disorder. Evidence to support this mechanism includes a neuroanatomical localization study that elucidated the anterior temporal cortex as a site frequently involved in strokes that are complicated by post-stroke depression (10). Of particular interest in this patient is the observation that this (anterior temporal) cortical territory comprised a significant portion of the damaged tissue, suggesting yet another feasible mechanism of behavioral change.

## 4. Conclusion

While the consideration of complex psychiatric and neurologic mechanisms can enhance the diagnostic approach in a patient like this, many questions remain unanswered. Therefore, clinicians should continue to investigate the fascinating and intricate array of pathophysiological mechanisms underlying alcohol use disorder in patients recovering from a stroke. Based on the evidence that already exists, the overall progression might be driven by several distinct processes, ranging from maladaptive psychological coping mechanisms to ischemic damage in specific neuroanatomic regions to exacerbations of underlying mood disorders. According to Occam’s razor, these seemingly separate processes might all be sequelae of one shared principal lesion. However, until that lesion is identified, there are numerous mechanisms, spanning multiple disciplines, that are worthwhile to explore as possible therapeutic targets.

## Data availability statement

The original contributions presented in the study are included in the article/Supplementary material, further inquiries can be directed to the corresponding author.

## Ethics statement

Ethical review and approval was not required for the study on human participants in accordance with the local legislation and institutional requirements. The patients/participants provided their written informed consent to participate in this study. Written informed consent was obtained from the participant/patient(s) for the publication of this case report.

## Author contributions

JZ and NK: substantial contributions to the conception or design of the work, or the acquisition, analysis, or interpretation of data for the work, drafting the work or revising it critically for important intellectual content, final approval of the version to be published, and agreement to be accountable for all aspects of the work in ensuring that questions related to the accuracy or integrity of any part of the work are appropriately investigated and resolved. All authors contributed to the article and approved the submitted version.

## Conflict of interest

The authors declare that the research was conducted in the absence of any commercial or financial relationships that could be construed as a potential conflict of interest.

## Publisher’s note

All claims expressed in this article are solely those of the authors and do not necessarily represent those of their affiliated organizations, or those of the publisher, the editors and the reviewers. Any product that may be evaluated in this article, or claim that may be made by its manufacturer, is not guaranteed or endorsed by the publisher.

## References

[1] Mavromatis LA Rosoff DB Cupertino RB Garavan H Mackey S Lohoff FW. Association between brain structure and alcohol use behaviors in adults: a Mendelian randomization and Multiomics study. JAMA Psychiat. (2022) 79:869–78. doi: 10.1001/jamapsychiatry.2022.2196, PMID: 35947372 PMC9366661

[2] Ayerbe L Ayis S Wolfe CD Rudd AG. Natural history, predictors and outcomes of depression after stroke: systematic review and meta-analysis. Br J Psychiatry. (2013) 202:14–21. doi: 10.1192/bjp.bp.111.107664, PMID: 23284148

[3] Sinyor D Amato P Kaloupek DG Becker R Goldenberg M Coopersmith H. Post-stroke depression: relationships to functional impairment, coping strategies, and rehabilitation outcome. Stroke. (1986) 17:1102–7. doi: 10.1161/01.str.17.6.1102, PMID: 3810708

[4] Attilia F Perciballi R Rotondo C Capriglione I Iannuzzi S Attilia ML. Alcohol withdrawal syndrome: diagnostic and therapeutic methods. Riv Psichiatr. (2018) 53:118–22. doi: 10.1708/2925.29413 29912213

[5] Holleck JL Merchant N Gunderson CG. Symptom-triggered therapy for alcohol withdrawal syndrome: a systematic review and meta-analysis of randomized controlled trials. J Gen Intern Med. (2019) 34:1018–24. doi: 10.1007/s11606-019-04899-7, PMID: 30937668 PMC6544709

[6] Kelly-Hayes M Beiser A Kase CS Scaramucci A D'Agostino RB Wolf PA. The influence of gender and age on disability following ischemic stroke: the Framingham study. J Stroke Cerebrovasc Dis. (2003) 12:119–26. doi: 10.1016/S1052-3057(03)00042-9 17903915

[7] Maaijwee NA Rutten-Jacobs LC Arntz RM Schaapsmeerders P Schoonderwaldt HC van Dijk EJ. Long-term increased risk of unemployment after young stroke: a long-term follow-up study. Neurology. (2014) 83:1132–8. doi: 10.1212/WNL.0000000000000817 25128177

[8] Corral M Holguín SR Cadaveira F. Neuropsychological characteristics of young children from high-density alcoholism families: a three-year follow-up. J Stud Alcohol. (2003) 64:195–9. doi: 10.15288/jsa.2003.64.195, PMID: 12713192

[9] Hasin DS Stinson FS Ogburn E Grant BF. Prevalence, correlates, disability, and comorbidity of DSM-IV alcohol abuse and dependence in the United States: results from the National Epidemiologic Survey on alcohol and related conditions. Arch Gen Psychiatry. (2007) 64:830–42. doi: 10.1001/archpsyc.64.7.830, PMID: 17606817

[10] Mayberg HS. Limbic-cortical dysregulation: a proposed model of depression. J Neuropsychiatry Clin Neurosci. (1997) 9:471–81. doi: 10.1176/jnp.9.3.471, PMID: 9276848

